# Investigation of the Biological Properties of (Hetero)Aromatic Thiosemicarbazones

**DOI:** 10.3390/molecules171113483

**Published:** 2012-11-14

**Authors:** Maciej Serda, Anna Mrozek-Wilczkiewicz, Josef Jampilek, Matus Pesko, Katarina Kralova, Marcela Vejsova, Robert Musiol, Alicja Ratuszna, Jaroslaw Polanski

**Affiliations:** 1 Department of Organic Chemistry, Institute of Chemistry, University of Silesia, PL-40006 Katowice, Poland; 2 Institute of Physics, University of Silesia, PL-40007 Katowice, Poland; 3 Department of Chemical Drugs, Faculty of Pharmacy, University of Veterinary and Pharmaceutical Sciences, Palackeho 1/3, CZ-61242 Brno, Czech Republic; 4 Research Institute for Pharmacy and Biochemistry, Lidicka 1879/48, CZ-60200 Brno, Czech Republic; 5 Department of Ecosozology and Physiotactics, Faculty of Natural Sciences, Comenius University, Mlynska dolina Ch-2, SK-84215 Bratislava, Slovakia; 6 Institute of Chemistry, Faculty of Natural Sciences, Comenius University, Mlynska dolina Ch-2, SK-84215 Bratislava, Slovakia; 7 Department of Clinical Microbiology, Charles University Medical School and Teaching Hospital, Sokolska 581, CZ-50005 Hradec Kralove, Czech Republic

**Keywords:** thiosemicarbazones, lipophilicity, photosynthetic electron transport inhibition, spinach chloroplasts, iron chelators, *in vitro* antifungal activity, *in vitro* anticancer activity

## Abstract

Two series of thiosemicarbazone-based iron chelators (twenty-seven compounds) were designed and synthesized using a microwave-assisted approach. Quinoline and halogenated phenyl were selected as parent scaffolds on the basis of a similarity search. The lipophilicity of the synthesized compounds was measured using HPLC and then calculated. Primary *in vitro* screening of the synthesized compounds was performed against eight pathogenic fungal strains. Only a few compounds showed moderate activity against fungi, and (*E*)-2-(quinolin-2-ylvinyl)*-N*,*N*-dimethylhydrazine-carbothioamide appeared to be more effective than fluconazole against most of the fungal strains tested. Antiproliferative activity was measured using a human colon cancer cell line (HCT-116). Several of the tested compounds showed submicromolar antiproliferative activity. Compounds were also tested for their activity related to the inhibition of photosynthetic electron transport (PET) in spinach (*Spinacia oleracea* L.) chloroplasts. The structure-activity relationships are discussed for all of the compounds.

## 1. Introduction

Iron is an element of great importance for virtually all living organisms. It is present in various biological processes such as metabolic pathways, reproduction or photosynthesis. On the basis of its importance, the iron-sulphur theory of the origin of life has been proposed [1,2]. Despite being so pervasive, iron rarely exists alone or in the form of simple cations. It is commonly chelated by larger organic molecules such as porphyrin. This form is present in hemoglobin or in most of the metabolic enzymes of cytochrome P_450_ [3]. Clearly, all living organisms have their ways of acquiring and managing such an important element. Any alteration of or damage to the iron metabolism system may lead to serious diseases. In humans, this may cause hemochromatosis or iron deficiency anemia [4]. As a target for therapy, iron is used mainly in the treatment of iron overload using iron chelators such as deferoxamine (DFO, also known as desferrioxamine B) [5]. More recently, iron chelators have been proposed for cancer treatment [5,6,7,8,9,10] because rapidly proliferating cancer cells have a higher demand for this element compared to the normal cells [5,11]. Although the exact mechanism of the anticancer activity of iron chelators remains unknown, several possible modes of action can be implemented, e.g., the inhibition of iron uptake [12] and the inhibition of ribonucleotide reductase (RR) [13] or the formation of reactive oxygen species (ROS) [14].

Other potential therapeutic or agricultural application of iron chelators may be derived from processes or pathways that are dependent on iron in pathological organisms. Fungi and bacteria require an appropriate amount of iron for correct growth, and apparently to develop their resistance to drugs [15,16]. Pathogenic microbes have evolved their unique way of acquiring this important metal from a host organism through the overexpression of Fe receptors or the utilization of siderophores [17]. If the iron level is reduced, fungi lose some of their virulence and have a decreased potency to invade the epithelium. Hence, lactoferrin and deferoxamine have been tested for their antifungal potency. Early attempts have consisted in the co-administration of lactoferrin with conventional antifungals such as amphotericin B [18,19]. DFO was tested as an ancillary drug with amphotericin B in antifungal therapy in a clinical trial [20,21]. The study was terminated prematurely due to an increased mortality in patients who had taken DFO when compared to those who had taken a placebo. However, this seems to be an effect of the more complex prerequisites connected with the unknown site of administration of DFO rather than with synergic toxicity. Nevertheless, this is a trigger to search for structurally new iron chelators with an antifungal activity. Several reports describing the synthesis and antifungal activity of thiosemicarbazones have been published [22,23,24,25,26]. Li *et al.* described a series of benzyl thiosemicarbazones whose substitution with a chlorine atom triggered the activity of mushroom tyrosinase [27]. Moreover, chlorine derivatives of benzaldehyde 4-phenyl-3-thiosemicarbazone and their cyclization products were tested against *Toxoplasma gondii* and several bacterial strains [28]. Some of these derivatives were more active than chloramphenicol and rifampicin.

**Figure 1:**
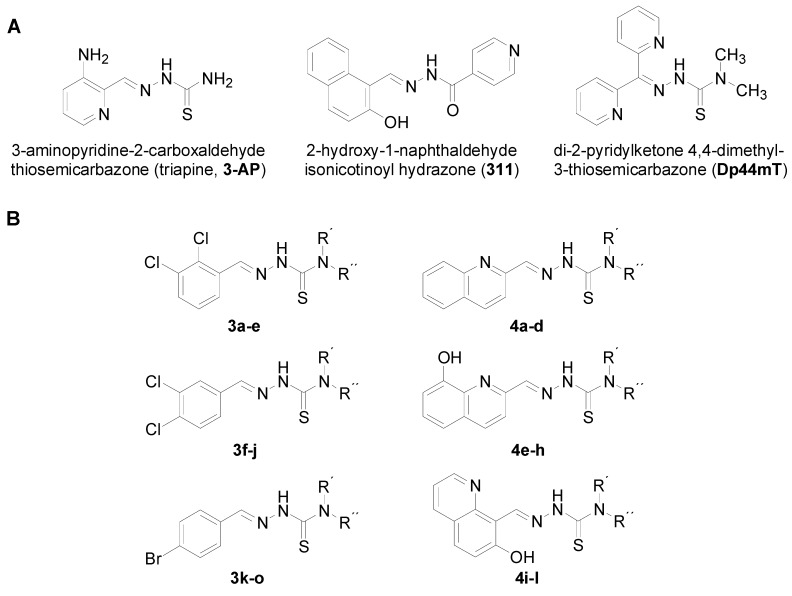
(**A**) Structures of known iron chelators 3-AP [5], 311 [35], Dp44mT [36,37]. (**B**) investigated benzylidenethiosemicarbazones **3a**–**o**, quinolinylvinylthiosemicarbazones **4a**–**l**.

In this paper, some disubstituted benzaldehyde thiosemicarbazones are described along with their anticancer and antifungal activity. These structures were tested on the basis of a former study on antifungal compounds [29,30,31,32,33] and quinolone-based thiosemicarbazones with anticancer activity [34]. Figure 1 illustrates the fragment-based similarity of the compounds investigated with known and effective iron chelators [5,35,36,37]. The design of compounds with polypharmacological activity was the aim of our investigation. Such compounds may be of great value in anticancer therapy where fungal infections are very common. Furthermore, these compounds, due to their simple and cheap synthesis, are suitable for agricultural use. However, antifungal agents such as azoles used for crop protection raise some concerns, e.g., triggering resistance [38] and have a negative impact on the environment. In fact, iron is also important in plants where it plays a crucial role in photosystems I and II as a part of ferredoxin [39] and cytochrome b559 [40,41,42,43]. Taking this aspect into account, thiosemicarbazone iron chelators may exert some unwanted effects on the green parts of plants. Thus, the compounds prepared for the inhibition of photosynthetic electron transport were also tested.

## 2. Results and Discussion

### 2.1. Chemistry

**Scheme 1:**
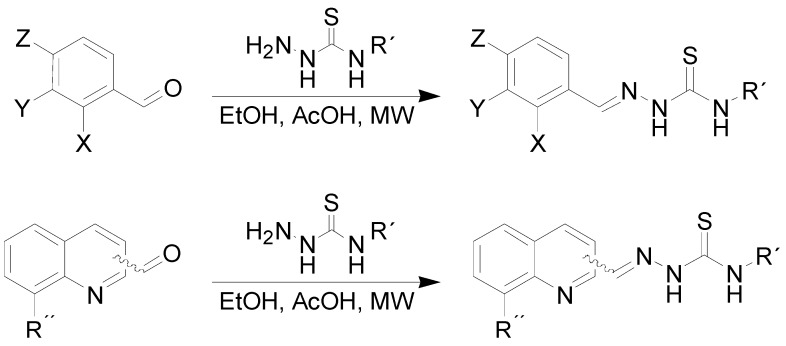
Synthesis of the discussed benzylidenethiosemicarbazones **3a**–**o** and quinolinylvinylthiosemicarbazones **4a**–**l**.

Compounds **3a**–**o** were obtained as shown in Scheme 1. Microwave irradiation was found to be helpful for enhancing the yield of the syntheses and the purity of the final products (the purity of all of the compounds was assessed using HPLC and was higher than 98%, while the overall average yield exceeded 80%). All of the synthesized compounds were in the *E*-configuration*,* which was confirmed using ^1^H-NMR spectroscopy (the signal of the NH group was in the 10–11 ppm range, in comparison to *Z*-isomer, which possesses a characteristic NH signal in the 14–15 ppm range), the COSY and NOESY experiments and by crystal structure as was reported recently [44]. Exemplary spectra are available in Supplementary Material for this article. 

### 2.2. Lipophilicity

**Figure 2:**
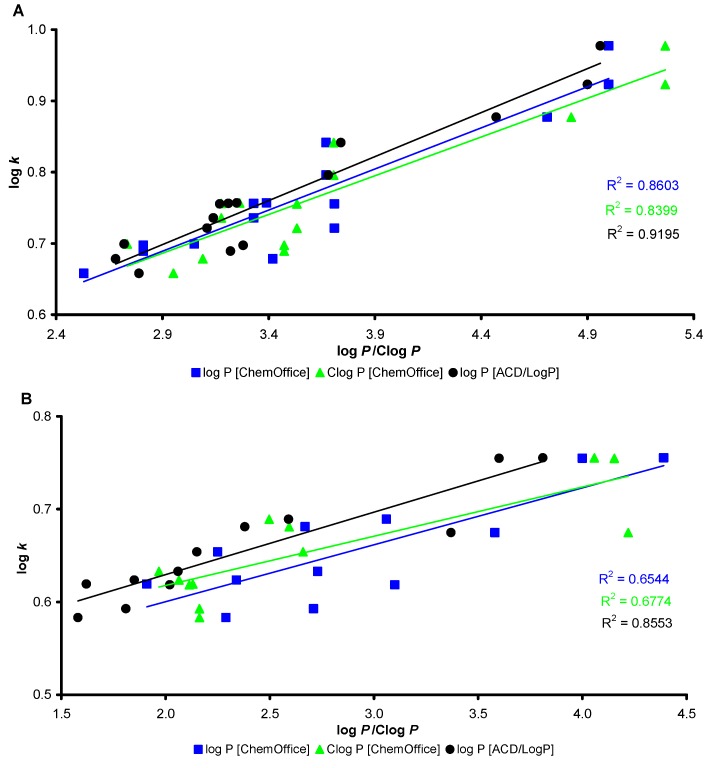
Comparison of log *P*/Clog *P* data calculated using two programs with log *k* values found experimentally. (**A**) match of calculated data with log *k* values of **3a**–**o** found experimentally; (**B**) match of calculated data with log *k* values of **4a**–**l** found experimentally.

Lipophilicity is a property that has a major effect on absorption, distribution, metabolism, excretion and toxicity properties as well as on pharmacological activity because drugs cross biological membranes through passive transport, which is strongly dependent on their lipophilicity. Lipophilicity has been studied and applied as an important drug property for decades. The lipophilicity of the compounds studied was determined using RP-HPLC as a capacity factor logarithm (log *k*) and calculated as log *P*/Clog *P* using two commercially available software programs (ChemOffice and ACD/LogP) [45,46]. The results for benzylidenethiosemicarbazones **3a**–**o** are shown in Table 1, they are summarized for quinolinylvinylthiosemicarbazones **4a**–**l** in Table 2 and are illustrated for both series in Figure 2.

Reversed phase high performance liquid chromatography (RP-HPLC) methods have become popular and are widely used for lipophilicity measurements. The general procedure is the measurement of the directly accessible retention time under isocratic conditions using varying amounts of methanol as an organic modifier in the mobile phase using RP columns and calculating the logarithm of the capacity factors (log *k*). Log *k* is the logarithm of the capacity factors in chromatographic approaches that is related to the partitioning of a compound between a mobile and a (pseudo-)stationary phase. Log *k* is used as the lipophilicity index converted to log *P* scale.

**Table 1:** Structure of aryl compounds **3a**–**o** and comparison of the calculated lipophilicities (log *P*/Clog *P*) with determined log *k* values.

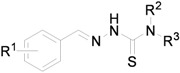						
Comp.	R^1^	R^2^	R^3^	log *k*	log *P*/Clog *P* ChemOffice	log *P* ACD/LogP
**3a**	2,3-Cl	H	H	0.6892	2.81/3.473	3.22 ± 0.38
**3b**	2,3-Cl	H	CH_3_	0.7357	3.33/3.177	3.14 ± 0.59
**3c**	2,3-Cl	CH_3_	CH_3_	0.7214	3.71/3.533	3.11 ± 0.60
**3d**	2,3-Cl	H	C_2_H_5_	0.7958	3.67/3.706	3.68 ± 0.59
**3e**	2,3-Cl	H	C_6_H_5_	0.9230	5.00/5.266	4.90 ± 0.59
**3f**	3,4-Cl	H	H	0.6974	2.81/3.473	3.28 ± 0.38
**3g**	3,4-Cl	H	CH_3_	0.7563	3.33/3.177	3.21 ± 0.59
**3h**	3,4-Cl	CH_3_	CH_3_	0.7555	3.71/3.533	3.17 ± 0.60
**3i**	3,4-Cl	H	C_2_H_5_	0.8415	3.67/3.706	3.74 ± 0.59
**3j**	3,4-Cl	H	C_6_H_5_	0.9774	5.00/5.266	4.96 ± 0.59
**3k**	4-Br	H	H	0.6580	2.53/2.952	2.79 ± 0.39
**3l**	4-Br	H	CH_3_	0.6993	3.05/2.734	2.72 ± 0.61
**3m**	4-Br	CH_3_	CH_3_	0.6784	3.42/3.090	2.68 ± 0.62
**3n**	4-Br	H	C_2_H_5_	0.7568	3.39/3.263	3.25 ± 0.61
**3o**	4-Br	H	C_6_H_5_	0.8772	4.71/4.823	4.47 ± 0.61

Commercially available chemical software does not resolve the various lipophilicity values of individual positional isomers, *etc*. The software calculates lipophilicity contributions according to different internal databases/libraries and uses different approaches. It can be concluded that for small, highly functionalized molecules with many heteroatoms, a number of intermolecular forces and intramolecular interactions are typical. The more of these interactions that can be expected, the less the predictability of common software can be, thus experimental lipophilicity determination is of great importance. Therefore, we decided to perform our measurements using the water-methanol system as the mobile phase. Log *k* derived from RP-HPLC retention factors and computational log *P* values are given in the manuscript and biological data are related to log *k* data because log *k* data specify lipophilicity within the series of compounds more precisely than the available chemical software.

The results obtained with all the compounds show that the experimentally-determined lipophilicities (log *k*) of the compounds discussed are relatively in accordance with the calculated values of compounds **3a**–**o** and **4a**–**l** as is shown in Figure 2. The minimum match of experimental (log *k*) versus calculated (log *P*) values was found for ChemOffice. Generally, benzylidenethiosemicarbazones **3a**–**o** showed a higher match (see Figure 2A) than quinolinylvinylthiosemicarbazones **4a**–**l** (see Figure 2B), which indicates inter- and intramolecular interactions in the last mentioned series.

**Table 2:** Structure of heteroaryl compounds **4a**–**l** and comparison of calculated lipophilicities (log *P*/Clog *P*) with determined log *k* values.

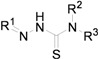						
Comp.	R^1^	R^2^	R^3^	log *k*	log *P*/Clog *P* ChemOffice	log *P* ACD/LogP
**4a**	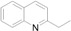	H	CH_3_	0.6329	2.73/1.968	2.06 ± 0.59
**4b**	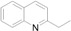	CH_3_	CH_3_	0.6184	3.10/2.114	2.02 ± 0.60
**4c**	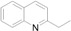	H,	C_2_H_5_	0.6892	3.06/2.497	2.59 ± 0.59
**4d**	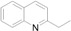	H	C_6_H_5_	0.7553	4.39/4.057	3.81 ± 0.59
**4e**	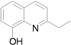	H	CH_3_	0.6236	2.34/2.064	1.85 ± 0.84
**4f**	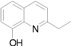	CH_3_	C_6_H_5_	0.5927	2.71/2.163	1.81 ± 0.85
**4g**	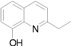	H	C_2_H_5_	0.6810	2.67/2.593	2.38 ± 0.84
**4h**	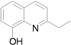	H	C_6_H_5_	0.7548	4.00/4.153	3.60 ± 0.84
**4i**	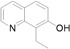	H	CH_3_	0.6193	1.91/2.131	1.62 ± 1.09
**4j**	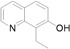	CH_3_	CH_3_	0.5831	2.29/2.163	1.58 ± 1.10
**4k**	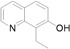	H	C_2_H_5_	0.6539	2.25/2.660	2.15 ± 1.09
**4l**	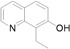	H	C_6_H_5_	0.6748	3.58/4.220	3.37 ± 1.09

Lipophilicity calculations of these small and highly functionalized molecules do not cover inter- and intramolecular interactions that can be found by experimental lipophilicity determination. As was expected, quinolinylvinylthiosemicarbazones **4a**–**l** showed a lower lipophilicity (log *k*) than benzylidene-thiosemicarbazones **3a**–**o**. Within the series of benzylidenethiosemicarbazones, the determined log *k* values increased as follows: 4-Br (**3k**–**o**) < 2,3-Cl (**3a**–**e**) < 3,4-Cl (**3f**–**j**), while within the series of quinolinylvinylthiosemicarbazones, the determined log *k* values increased in the following order: 7-hydroxyquinolin-8-yl (**4i**–**l**) < 8-hydroxyquinolin-2-yl (**4e**–**h**) < quinoline-2-yl (**4a**–**d**). The influence of R^2^ and R^3^ substituents on lipophilicity is as follows: 2H < 2CH_3_ < H, CH_3_ < H, C_2_H_5_ < H, C_6_H_5_. Based on the above-mentioned data, it can be assumed that compounds **4e**–**l** are possible tautomeric isomers. This seems to be in agreement with our former findings [47]. Therefore, it can be concluded that log *k* values specify lipophilicity within individual series of the compounds studied more precisely than the calculated log *P*/Clog *P* data.

### 2.3. Biological Activities

The compounds under investigation can be divided into two groups based on their chemical structure: Group 1 included benzylidenethiosemicarbazones **3a**–**o** and Group 2 contained quinolinylvinylthiosemicarbazones **4a**–**l**. The compounds showed a wide range of biological activities and some interesting structure-activity relationships were observed. All of the results are summarized in Table 3.

#### 2.3.1. Inhibition of Photosynthetic Electron Transport (PET) in Spinach Chloroplasts

The activity of all of the evaluated derivatives related to the inhibition of photosynthetic electron transport (PET) in spinach (*Spinacia oleracea* L.) chloroplasts was moderate or rather low relative to the standard (see Table 3). Diuron^®^ (DCMU), a marketed herbicide with its activity mechanism directed to photosystem II, was used as the standard. The compounds within Group 2 could not be evaluated for their structure-activity relationships because the majority of these compounds showed no PET-inhibiting activity. (*E*)-2-(3,4-Dichlorobenzylidene)-*N*-methylhydrazinecarbothioamide (**3g**) expressed the highest PET-inhibiting activity (IC_50_ = 135.6 µmol/L) within Group 1.

**Table 3:** IC_50_ [μmol/L] values related to PET inhibition in spinach chloroplasts of the substituted thiosemicarbazones in comparison with 3-(3,4-dichlorophenyl)-1,1-dimethylurea (DCMU) as the standard; *in vitro* antifungal activity (IC_80_/IC_50_ [μmol/L]) of compounds compared to fluconazole (FLU) as the standard; *in vitro* antiproliferative activity IC_50_ [μmol/L] of compounds compared to doxorubicin as the standard. ND = not determined due to precipitation during the experiment or interaction with 2,6-dichlorophenol-indophenol (DCPIP).

Comp.	PET IC_50_	^1,2^ MIC (^a^ IC_80_ / ^b^ IC_50_) [µmol/L]								HCT-116 IC_50_
		CA ^a^	CT ^a^	CK ^a^	CG ^a^	TB ^a^	AF ^b^	AC ^b^	TM ^b^	
		24 h	24 h	24 h	24 h	24 h	24 h	24 h	72 h	
		48 h	48 h	48 h	48 h	48 h	48 h	48 h	120 h	
**3a**	ND	>125	>125	>125	>125	>125	>125	>125	>125	41.5 ± 1.7 3
**3b**	170.1	>500	>500	>500	>500	>500	>500	>500	>500	>60
**3c**	ND	>125	>125	>125	>125	>125	>125	>125	>125	-
**3d**	ND	>500	>500	>500	>500	>500	>500	>500	>500	58.5 ± 0.2 3
**3e**	499.3	>125	>125	>125	>125	>125	>125	>125	>125	>60
**3f**	283.3	>500	>500	>500	>500	>500	>500	>500	>500	46.0 ± 0.9
**3g**	**135.6**	>125	>125	>125	>125	>125	>125	>125	>125	55.5 ± 6.2
**3h**	329.3	**15.62**	**500**	**500**	**500**	**125**	>500	>500	**31.25**	47.0 ± 1.3
		**31.25**	**500**	**500**	**500**	**500**	>500	>500	**62.5**	
**3i**	280.2	>500	>500	>500	>500	>500	>500	>500	>500	48.5 ± 2.8
**3j**	425.9	>125	>125	>125	>125	>125	>125	>125	>125	>60
**3k**	586.4	**500**	>500	>500	>500	**500**	>500	>500	>500	>60
		**500**	>500	>500	>500	**500**	>500	>500	>500	
**3l**	ND	>500	>500	>500	>500	>500	>500	>500	>500	>60
**3m**	594.6	**62.5**	>500	>500	>500	**15.62**	>500	>500	**62.5**	47.5 ± 2.4
		**250**	>500	>500	>500	**62.5**	>500	>500	**62.5**	
**3n**	ND	>500	>500	>500	>500	>500	>500	>500	>500	>60 3
**3o**	ND	>500	>500	>500	>500	>500	>500	>500	>500	>60
**4a**	ND	>500	>500	>500	>500	>500	>500	>500	>500	>25
**4b**	1368	**1.95**	**1.95**	**3.9**	**1.95**	**1.95**	**3.9**	**3.9**	**3.9**	4.86 ± 1.48 3
		**1.95**	**7.81**	**3.9**	**1.95**	**1.95**	**3.9**	**3.9**	**3.9**	
**4e**	302	>125	>125	>125	>125	>125	>125	>125	>125	1.71 ± 0.34 3
**4f**	ND	>500	>500	>500	>500	>500	>500	>500	>500	-
**4h**	ND	>125	>125	>125	>125	>125	>125	>125	>125	24.97 ± 4.29 3
		>125	>125	>125	>125	>125	>125	>125	>125	
**4i**	ND	>500	>500	>500	>500	>500	>500	>500	>500	>25 3
		>500	>500	>500	>500	>500	>500	>500	>500	
**4j**	520.4	**125**	>250	>250	**125**	**125**	>250	>250	**125**	20.75 ± 5.34 3
		>250	>250	>250	**125**	>250	>250	>250	**125**	
**4k**	ND	>500	>500	>500	**62.5**	>500	>500	>500	**62.5**	>25 3
		>500	>500	>500	**500**	>500	>500	>500	**62.5**	
**4l**	ND	**31.25**	**125**	**31.25**	**15.62**	**15.62**	**125**	**15.62**	**15.62**	16.28 ± 1.69 3
		**62.5**	**125**	**125**	**31.25**	**125**	**125**	**62.5**	**15.62**	
**DCMU**	**1.9**	-	-	-	-	-	-		-	-
**FLU**	-	**0.06**	**0.12**	**3.91**	**0.98**	**0.24**	>125	>125	**1.95**	-
		**0.12**	>125	**15.62**	**3.91**	**0.48**	>125	>125	**3.91**	
**DXR**	-	-	-	-	-	-	-	-	-	10 ± 1.1

^1^ The MIC determination was performed according to the CLSI reference protocol: ^a^ M27-A2 for yeasts (IC_80_ value) and ^b^ M38-A for moulds (IC_50_ value); CA = *Candida albicans*, CT = *Candida tropicalis*, CK = *Candida krusei*, CG = *Candida glabrata*, TB = *Trichosporon beigelii*, AF = *Aspergillus fumigatus*, AC = *Absidia corymbifera*, and TM = *Trichophyton mentagrophytes*. ^2^ All compounds were tested for short and long term activity. When inactive only one value is presented. ^3^ Synthesis and anticancer activity described elsewhere [34,44]. HCT-116 human colon cancer cells, DXR- doxorubicin as the standard.

**Figure 3:**
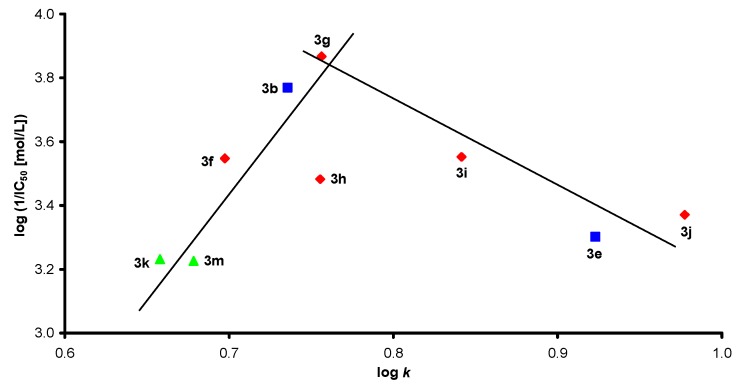
Relationships between PET inhibition log (1/IC_50_) [mol/L] in spinach chloroplasts and lipophilicity of studied benzylidenethiosemicarbazones **3a**–**o**.

Figure 3 illustrates the dependence of the PET-inhibiting activity, expressed by the negative logarithm of the log (1/IC_50_[mol/L]) value on the lipophilicity, expressed as log *k*, of benzylidenethiosemicarbazones **3a**–**o**. Although the dependence seems to be clear, it is not possible to draw a simple relationship. The regression with parabolic function did not provide a good correlation. This suggests that lipophilicity is only one of the important factors. Nevertheless, despite the relatively low inhibitory activity of the compounds studied, the correlations between log (1/IC_50_) and the lipophilicity showed a bilinear course: in the range of log *k* = 0.6580 (**3k**) to log *k* = 0.7563 (**3g**) PET-inhibiting activity linearly increased with increasing compound lipophilicity; however, with a further increase in lipophilicity, the inhibitory activity declined. Generally, 4-Br-benzylidenethiosemi-carbazones **3k**–**o** showed the worst PET inhibition. Disubstitution by 3,4-Cl of the benzylidene ring seems to be more advantageous than disubstitution by 2,3-Cl of the benzylidene moiety. It can be stated that within the 3,4-Cl-benzylidenethiosemicarbazones **3f**–**j** *N*,*N*-disubstitution caused a decrease in PET inhibition (compare **3g**/**3h**). Based on these facts, it can be concluded that PET-inhibiting activity would probably be negatively influenced by bulky or long-chain alkyls or by the substitution of the second hydrogen of the terminal amino moiety.

#### 2.3.2. *In Vitro* Antifungal Susceptibility Testing

The antifungal activity of the compounds studied was tested against several pathogenic fungi and the results of the screening are shown in Table 3. The values are correlated to the standard drug fluconazole. The majority of the compounds studied appeared to be inactive against the fungal strains tested. Nevertheless, some of them, **3h**, **3m**, and **4l**, showed moderate activity, especially against *Candida albicans*, *Trichosporon beigelii*, and *Trichophyton mentagrophytes*. (*E*)-2-(Quinolin-2-ylvinyl)*-N*,*N*-dimethylhydrazinecarbothioamide (**4b**) expressed the strongest activity among all of the compounds tested. Compound **4b** was more active than fluconazole against *Aspergillus fumigatus* and *Absidia corymbifera*. Furthermore, this compound showed an interesting pattern of long terminal activity and was active at a comparable level for 24 h and 48 h protocols. The (*Candida tropicalis* and *Candida krusei*) activity of fluconazole decreased significantly for some fungi.

As most compounds showed only a moderate or no activity, no thorough structure-activity relationships could be established. According to the results presented in Table 3, it can be concluded that compounds with a quinoline nucleus, *i.e.*, quinolinylvinylthiosemicarbazones (Group 2) seem to be more effective antifungals than Group 1, *i.e.*, benzylidenethiosemicarbazones. Based on recent results [32], it was expected that 8-hydroxyquinolinylvinylthiosemicarbazones would possess a higher antifungal activity than antifungal tests showed. It can be stated, contrary to PET-inhibiting activity, that *N*,*N*-disubstitution of the terminal amino moiety by two methyl groups is crucial for a high antifungal effect. This fact corresponds to results obtained by Opletalova *et al.* [26].

#### 2.3.3. *In Vitro* Antiproliferative Activity

The antiproliferative activity of all of the compounds was examined against the human colon cancer (HCT-116) cell line using an MTS assay. The HCT-116 p53 wild type cell line was chosen on the basis of our former experience and some results on the activity of quinoline-based thiosemicarbazones that was published recently. Namely, compounds **4a–l** have been tested previously for their wide-ranging anticancer activity [34]. The antiproliferative activity of the compounds evaluated was assessed using Doxorubicin as a reference compound. In general, quinoline-based thiosemicarbazones **4** seem to have a much better anticancer activity than halogenophenyl compounds **3**. Thus, the quinoline nucleus can be defined as being crucial for antiproliferative activity. A more detailed analysis of the anticancer activity of quinoline-based thiosemicarbazone is available [34]. Benzylidenethiosemicarbazones were much less active against the cells tested. Compound **3a** appeared to be the most active in this assay. Nevertheless, some more general conclusions can be drawn from the gathered data. Dichlorosubstituted compounds are in general more active than monobromo equivalents. The free amino group or substitution pattern with small substituents such as methyl or ethyl are favorable for activity. According to our former results the chelating ability of the compounds may be one of the prerequisites of the antiproliferative activity. In this study we have measured the UV-VIS spectra of the compounds in presence of various concentration of Fe^3+^ cations to observe its chelating potency (supplementary material). Isosbestic points are observable in case of more active compounds like **4a–l** series while in **3a–o** only minor or no interaction between ligand and metal can be noticed. Compound **4b** one of the most active against HCT-116 cells and in antifungal assays gave clear isosbestic points at 375 nm. However as **4j** give also two points at 330 and 385 nm, iron chelating ability should be regarded only as one of the factor influencing the biological activity.

## 3. Experimental

### 3.1. General

All reagents were purchased from Sigma-Aldrich (St. Louis, MO, USA) or Princeton Chemicals Ltd. (Luton, Bedfordshire, UK). Kieselgel 60, 0.040–0.063 mm (Merck, Darmstadt, Germany) was used for the column chromatography. Syntheses were performed in a CEM DISCOVERY microwave reactor (Matthews, NC, USA) with temperature and pressure control. TLC experiments were performed on alumina-backed silica gel 40 F_254_ plates (Merck, Darmstadt, Germany). The plates were illuminated under UV (254 nm) and evaluated in an iodine vapor. The melting points were determined on an Optimelt MPA100 instrument (SRS, USA) and are uncorrected. Infrared (IR) spectra were recorded on a Smart MIRacle™ ATR ZnSe for Nicolet™ Impact 410 FT-IR spectrometer (Thermo Fisher Scientific, Waltham, MA, USA). The spectra were obtained by the accumulation of 256 scans with 2 cm^−1^ resolution in the region of 4000–600 cm^−1^. All ^1^H and ^13^C-NMR spectra were recorded on a Bruker Avance III 400 MHz FT-NMR spectrometer (400 MHz for ^1^H and 100 MHz for ^13^C, BrukerBioSpin Comp., Karlsruhe, Germany). Chemicals shifts are reported in ppm (δ) using internal Si(CH_3_)_4_ as the reference with diffuse, easily exchangeable signals being omitted. HR-MS (EI) analysis was performed on a Finnigan MAT95 spectrometer (ThermoFinnigan, San Jose, CA, USA) for all new compounds.

### 3.2. Synthesis

#### 3.2.1. General Procedure for Synthesis of Thiosemicarbazones **3a–o**

Equimolar quantities of an appropriate thiosemicarbazide and benzaldehyde derivative were mixed and 2 drops of glacial acetic acid were added. The resulting mixture was heated in a microwave reactor at 85 °C for 12 min (max microwave power 75 W). After cooling, the precipitated solids were filtered off, washed with ether and crystallized from methanol. The compounds **3a**–**o** studied are presented in Table 1.

*(E)-2-(2,3-Dichlorobenzylidene)hydrazinecarbothioamide* (**3a**). Yield: 78%, ^1^H-NMR (*d*_6_-DMSO, 400 MHz, ppm): 11.69 (bs, 1H, NH), 8.49 (s, 1H, CH), 8.36 (s, 1H, NH), 8.30 (d, *J* = 8.0 Hz, 1H, ArH), 8.18 (bs, 1H, NH), 7.66 (d, *J* = 7.9 Hz, 1H, ArH), 7.38 (t, *J* = 8.0 Hz, 1H, ArH). ^13^C-NMR (*d*_6_-DMSO, 100 MHz, ppm): 178.8, 138.2, 134.2; 132.6, 131.6, 131.2, 128.5, 126.5. MP: 226–227 °C HR-MS (EI): 246.9745 (calc. for C_8_H_7_Cl_2_N_3_S: 246.9738).

*(E)-2-(2,3-Dichlorobenzylidene)-N-methylhydrazinecarbothioamide* (**3b**). Yield: 74%, ^1^H-NMR (*d*_6_-DMSO, 400 MHz, ppm): 11.76 (s, 1H, NH), 8.68 (d, *J* = 4.4 Hz, 1H, NH), 8.49 (s, 1H, CH), 8.29 (d, *J* = 8.0 Hz, 1H, ArH), 7.67 (d, *J* = 7.9 Hz, 1H, ArH), 7.41 (t, *J* = 7.9 Hz, 1H, ArH), 3.02 (d, *J* = 4.5 Hz, CH_3_). ^13^C-NMR (*d*_6_-DMSO, 100 MHz, ppm): 178.5, 137.7, 134.5, 132.5, 131.4, 131.3, 128.4, 126.2, 31.4. MP: 223–224 °C, HR-MS (EI): 260.9891 (calc. for C_9_H_9_Cl_2_N_3_S: 260.9894).

*(E)-2-(2,3-Dichlorobenzylidene)-N,N-dimethylhydrazinecarbothioamide* (**3c**). Yield: 69%, ^1^H-NMR (*d*_6_-DMSO, 400 MHz, ppm): 11.27 (s, 1H, NH), 8.64 (s, 1H, CH), 7.92 (dd, J_1_ = 7.9 Hz, J_2_ = 1.4 Hz, 1H, ArH), 7.66 (dd, J_1_ = 7.9 Hz, J_2_ = 1.5 Hz, 1H, ArH), 7,42 (t, *J* = 8.0 Hz, 1H, ArH), 3.31 (s, 6H, CH_3_). ^13^C-NMR (*d*_6_-DMSO, 100 MHz, ppm): 180.8, 140.0, 134.7, 132.7, 131.5, 131.0, 128.8, 125.7, 42.4. MP: 145–146 °C, HR-MS (EI): 275.0047 (calc. for C_10_H_11_Cl_2_N_3_S: 275.0051).

*(E)-2-(2,3-Dichlorobenzylidene)-N-ethylhydrazinecarbothioamide* (**3b**). Synthesis and more detailed analysis of the structure is available [44]. Yield: 67%, ^1^H-NMR (*d*_6_-DMSO, 400 MHz, ppm): 11.70 (s, 1H, NH), 8.70 (m, 1H, NH), 8.49 (s, 1H, CH), 8.31 (d, *J* = 7.9 Hz, 1H, ArH), 7.67(d, *J* = 7.9 Hz, 1H, ArH), 7.41 (t, *J* = 7.9 Hz, 1H, ArH), 3.70–3.52 (m, 2H, CH_2_), 1.16 (t, *J* = 7.0 Hz, 3H, CH_3_). ^13^C-NMR (*d*_6_-DMSO, 100 MHz, ppm): 177.4, 137.6, 134.4, 132.5, 131.5, 131.3, 128.5, 126.02, 38.9, 14.7. MP: 213–214 °C HR-MS (EI): 275.0054 (calc. for C_10_H_11_Cl_2_N_3_S: 275.0051), IR: 3349 (ν_NH_), 3142 (ν_PhH_), 2983 (ν_CH_), 1584 (ν_C=N_), 1542 (ν_C-N_), 1520 (ν_CH_), 1452 (ν_CH/NH_), 1416 (ν_CH3_), 1311 (ν_CNH_), 1264 (ν_Ν−__C_/δ_NH_), 1160(ν_N-N_), 1108 (ω_ΝΗ_), 1042 (ρ_NH_), 929 , 906 (δ_Ν__CS_), 782 (ν_CS_), 737, 704 (ring), 626 (δ_Ν−__C-__N_). UV-VIS (methanol; logε): 323.0 (3.96), 237.0 (3.67), 204.0 (3.91).

*(E)-2-(2,3-dichlorobenzylidene)-N-phenylhydrazinecarbothioamide* (**3b**). Yield: 79%, ^1^H-NMR (*d*_6_-DMSO, 400 MHz, ppm): 12.08 (bs, 1H, NH), 10.26 (s, 1H, NH), 8.62 (s, 1H, CH), 8.47 (d, *J* = 9.3 Hz, 1H, ArH), 7.68 (t, *J* = 7.1 Hz, 1H, ArH), 7.55 (d, *J* = 7.7 Hz, 2H, ArH), 7.43–7.37 (m, 3H, ArH), 7.23 (t, *J* = 7.6 Hz, 1H, ArH). ^13^C-NMR (*d*_6_-DMSO, 100 MHz, ppm): 176.9, 139.5, 138.9, 134.3, 132.6, 131.8, 131.5, 128.6, 126.8, 126.6, 126.0. MP: 203–204 °C, HR-MS (EI): 323.0066 (calc. for C_14_H_11_Cl_2_N_3_S: 323.0051).

*(E)-2-(3,4-Dichlorobenzylidene)hydrazinecarbothioamide* (**3f**). Synthesis and more detailed analysis of the structure is available [44]. Yield: 85%, ^1^H-NMR (*d*_6_-DMSO, 400 MHz, ppm): 11.56 (s, 1H, NH), 8.28 (s, 1H, NH), 8.24 (d, *J* = 1.7 Hz, 1H, ArH), 8.00 (s, 1H, CH), 7.72 (dd, *J* = 8.4, 1.8 Hz, 1H, ArH), 7.64 (d, *J* = 8.3 Hz, 1H, ArH). ^13^C-NMR (*d*_6_-DMSO, 100 MHz, ppm): 178.7, 139.9, 135.6, 132.3, 132.3, 131.2, 128.7, 128.2. MP: 205–206 °C [34]. IR: 3395, 3256 (ν_NH_), 3154 (ν_PhH_), 2992 (ν_CH_), 1596 (δ_NH2_), 1538 (ν_C=N_), 1471 (δ_CH/NH_), 1291 (ν_Ν−Χ_/δ_NH_), 1129 (ν_N-N_), 1100 (ω_ΝΗ_), 1065 (ρ_NH_), 1031(ν_ring_), 938(δ_CH_), 870, 823 (ν_CS_), 625 (δ_Ν−__C-N_). UV-VIS (MeOH; log ε): 320.0 (4.01), 239.0 (3.63), 203.0 (3.92).

*(E)-2-(3,4-Dichlorobenzylidene)-N-methylhydrazinecarbothioamide* (**3f**). Yield: 89% ^1^H-NMR (*d*_6_-DMSO, 400 MHz, ppm): 11.63 (s, 1H, NH), 8.68 (d, *J* = 3.8 Hz, 1H, NH), 8.20 (s, 1H, CH), 8.00 (s, 1H, ArH), 7.69 (dd, J_1_ = 23.9, J_2_ = 8.3 Hz, 2H), 3.03 (d, *J* = 4.3 Hz, 3H). ^13^C-NMR (*d*_6_-DMSO, 100 MHz, ppm): 178.4, 139.4, 135.7, 132.2, 132.2, 131.2, 128.5, 128.1, 31.62. MP: 202–203 °C. HR-MS (EI): 260.9902 (calc. for C_9_H_9_Cl_2_N_3_S: 260.9894).

*(E)-2-(3,4-Dichlorobenzylidene)-N,N-dimethylhydrazinecarbothioamide* (**3h**). Yield: 79% ^1^H-NMR (*d*_6_-DMSO, 400 MHz, ppm): 11.12 (s, 1H, NH), 8.17 (s, 1H, CH), 7.86 (d, *J* = 1.7 Hz, 1H, ArH), 7.70–7.63 (m, 1H, ArH), 3.30 (s, 6H, CH_3_). ^13^C-NMR (*d*_6_-DMSO, 100 MHz, ppm): 181.0, 141.5, 136.0, 132.1, 132.0, 131.5, 128.4, 127.0, 42.5. MP: 138–139 °C HR-MS (EI): 275.0048 (calc. for C_10_H_11_Cl_2_N_3_S: 275.0051).

*(E)-2-(3,4-Dichlorobenzylidene)-N-ethylhydrazinecarbothioamide* (**3i**). Yield: 83%, ^1^H-NMR (*d*_6_-DMSO, 400 MHz, ppm): 11.56 (s, 1H, NH), 8.73 (t, *J* = 5.7 Hz, 1H, NH), 8.19 (d, *J* = 1.7 Hz, 1H, ArH), 8.00 (s, 1H, CH), 7.78–7.70 (m, 1H, ArH), 7.67 (d, *J* = 8.3 Hz, 1H), 3.61 (m, 2H, CH_2_), 1.16 (t, *J* = 7.1 Hz, 3H, CH_3_). ^13^C-NMR (*d*_6_-DMSO, 100 MHz, ppm): 177.3, 139.5, 135.6, 132.2, 132.2, 131.3, 128.6, 128.1, 38.8, 15.0. MP: 177–178 °C, HR-MS (EI): 275.0058 (calc. for C_10_H_11_Cl_2_N_3_S: 275.0051).

*(E)-2-(3,4-Dichlorobenzylidene)-N-phenylhydrazinecarbothioamide* (**3j**). Yield: 86%, ^1^H-NMR (*d*_6_-DMSO, 400 MHz, ppm): 11.96 (s, 1H, NH), 10.27 (s, 1H, NH), 8.35 (s, 1H, NH), 8.12 (s, 1H, Ar), 7.82 (dd, *J* = 8.4, 1.7 Hz, 1H, ArH), 7.68 (d, *J* = 8.3 Hz, 1H, ArH), 7.53 (d, *J* = 7.7 Hz, 2H, Ph), 7.39 (t, *J* = 7.8 Hz, 2H, Ph), 7.24 (t, *J* = 7.4 Hz, 1H, Ph). ^13^C-NMR (*d*_6_-DMSO, 100 MHz, ppm): 176.9, 140.6, 139.5, 135.4, 132.5, 132.3, 131.2, 128.9, 128.6, 126.9, 126.1. MP: 200–201 °C [28].

*(E)-2-(4-Bromobenzylidene)hydrazinecarbothioamide* (**3k**). Yield: 69% ^1^H-NMR (*d*_6_-DMSO, 400 MHz, ppm): 11.48 (s, 1H, NH), 8.23 (s, 1H, NH), 8.07 (s, 1H, NH), 8.02 (s, 1H, CH), 7.77 (d, *J* = 8.5 Hz, 2H, ArH), 7.59 (d, *J* = 8.5 Hz, 2H, ArH). ^13^C-NMR (*d*_6_-DMSO, 100 MHz, ppm): 178.6, 141.4, 134.0, 132.1, 129.6, 123.5. MP: 222 °C [48].

*(E)-2-(4-bromobenzylidene)-N-methylhydrazinecarbothioamide* (**3l**). Yield: 72%; ^1^H-NMR (*d*_6_-DMSO, 400 MHz, ppm): 11.54 (s, 1H, NH), 8.57 (d, *J* = 4.0 Hz, 1H), 8.01 (s, 1H, CH), 7.77 (d, *J* = 8.3 Hz, 2H, ArH), 7.61 (d, *J* = 8.4 Hz, 2H, ArH), 3.02 (d, *J* = 4.4 Hz, 3H, CH_3_). ^13^C-NMR (*d*_6_-DMSO, 100 MHz, ppm): 178.3, 140.8, 134.1, 132.0, 129.4, 123.4, 31.2. MP: 204–205 °C [49].

*(E)-2-(4-bromobenzylidene)-N,N-dimethylhydrazinecarbothioamide* (**3m**). Yield: 61%, ^1^H-NMR (*d*_6_-DMSO, 400 MHz, ppm): 11.00 (s, 1H, NH), 8.17 (s, 1H, CH), 7.71–7.45 (m, 4H, ArH), 3.29 (s, 6H, CH_3_). ^13^C-NMR (*d*_6_-DMSO, 100 MHz, ppm): 181.0, 143.0, 134.4, 132.2, 129.0, 123.1, 42.6. MP: 150–151 °C [50].

*(E)-2-(4-Bromobenzylidene)-N-ethylhydrazinecarbothioamide* (**3n**). Synthesis and more detailed analysis of the structure is available [44]. Yield: 78%, ^1^H-NMR (*d*_6_-DMSO, 400 MHz, ppm): 11.47 (s, 1H, NH), 8.61 (t, *J* = 5.8 Hz, 1H, NH), 8.02 (s, 1H, CH), 7.77 (d, *J* = 8.5 Hz, 2H, ArH), 7.61 (d, *J* = 8.5 Hz, 2H, ArH), 3.63–3.56 (m, *J* = 7.0 Hz, 2H, CH_2_), 1.15 (t, *J* = 7.1 Hz, 3H, ArH). ^13^C-NMR (*d*_6_-DMSO, 100 MHz, ppm): 177.2, 140.9, 134.1, 132.0, 129.6, 123.4, 38.8, 15.0. MP: 199–200 °C, HR-MS(EI): 284.9944 (calc. for C_10_H_12_BrN_3_S Exact Mass: 284,9935), IR: 3363 (ν_NH_), 3139 (ν_PhH_), 2980 (ν_CH_), 1589 (ν_C=N_), 1538 (ν_C-N_), 1522 (δ_CH_), 1485 (δ_CH/NH_), 1397 (δ_CNH_), 1293 (ν_Ν−__C_/δ_NH_), 1104 (ν_N-N_), 1081 (ω_ΝΗ_), 1066 (ρ_NH_), 1006 (ν_ring_), 921 (δ_CH_), 816 (ν_CS_), 623 (δ_Ν−__C-N_). UV-VIS (MeOH, log ε): 330 (sh), 319.5 (3.98), 233.0 (3.65), 218 (sh), 202.5 (3.91).

*(E)-2-(4-bromobenzylidene)-N-phenylhydrazinecarbothioamide* (**3o**). Yield: 68%, ^1^H-NMR (*d*_6_-DMSO, 400 MHz, ppm): 11.88 (s, 1H, NH), 10.17 (s, 1H, NH), 8.13 (s, 1H, CH), 7.89 (d, *J* = 8.5 Hz, 2H, ArH), 7.63 (d, *J* = 8.5 Hz, 2H, ArH), 7.56 (d, *J* = 7.6 Hz, 2H, Ph), 7.38 (t, *J* = 7.8 Hz, 2H, Ph), 7.22 (t, *J* = 7.4 Hz, 1H, Ph). ^13^C-NMR (*d*_6_-DMSO, 100 MHz, ppm):176.6, 142.0, 139.5, 133.9, 132.1, 130.0, 128.5, 126.5, 125.9, 123.7. MP: 197–198 °C [49].

#### 3.2.2. General Procedure for the Synthesis of Thiosemicarbazones **4a–l**

All quinoline-based thiosemicarbazones were prepared according to a recently published procedure [34]. Equimolar quantities of an appropriate thiosemicarbazide and benzaldehyde derivative were mixed and 2 drops of glacial acetic acid were added. The resulting mixture was heated in a microwave reactor at 85 °C for 12 min (max microwave power 75 W). After cooling, the precipitated soli were filtered and washed with ether and crystallized from methanol. The compounds **4a**–**l** studied are presented in Table 2. 

### 3.3. Lipophilicity Determination Using HPLC (Capacity Factor k/Calculated log k)

A Waters Alliance 2695 XE HPLC separation module and a Waters Photodiode Array Detector 2996 (Waters Corp., Milford, MA, USA) were used. A Symmetry^®^ C_18_ 5 μm, 4.6 × 250 mm, Part No. WAT054275 (Waters Corp.) chromatographic column was used. The HPLC separation process was monitored using Empower™ 2 Chromatography Data Software, Waters 2009 (Waters Corp.). A mixture of MeOH p.a. (55%) and H_2_O-HPLC-Mili-Q Grade (45%) was used as a mobile phase. The total flow of the column was 0.9 mL/min, injection volume 30 μL, column temperature 30 °C and sample temperature 10 °C. A detection wavelength of 210 nm was chosen. A KI methanolic solution was used for the determination of dead time (t_D_). Retention times (t_R_) were measured in minutes. The capacity factors *k* were calculated using the Empower™ 2 Chromatography Data Software according to the formula *k* = (t_R_ − t_D_)/t_D_, where t_R_ is the retention time of the solute, whereas t_D_ denotes the dead time obtained using an unretained analyte. Log *k*, calculated from the capacity factor *k*, is used as the lipophilicity index converted to log *P* scale. The log *k* values of the individual compounds are shown in Table 1 and Table 2.

### 3.4. Lipophilicity Calculations

Log *P*, *i.e.*, the logarithm of the partition coefficient for *n-*octanol/water, was calculated using the programs ChemOffice [45] and ACD/LogP [46]. Clog *P* values (the logarithm of *n*-octanol/water partition coefficient based on established chemical interactions) were generated using ChemOffice software [45]. The results are shown in Table 1 and Table 2.

### 3.5. Study of the Inhibition Photosynthetic Electron Transport (PET) in Spinach Chloroplasts

Chloroplasts were prepared from spinach (*Spinacia oleracea* L.) according to Masarovicova and Kralova [51]. The inhibition of the photosynthetic electron transport (PET) in spinach chloroplasts was determined spectrophotometrically (Genesys 6, Thermo Fisher Scientific, Waltham, MA, USA) using an artificial electron acceptor 2,6-dichlorophenol-indophenol (DCIPP) according to Kralova *et al*. [52] and the rate of photosynthetic electron transport was monitored as a photoreduction of DCPIP. The measurements were carried out in a phosphate buffer (0.02 mol/L, pH 7.2) containing sucrose (0.4 mol/L), MgCl_2_ (0.005 mol/L) and NaCl (0.015 mol/L). The chlorophyll content was 30 mg/L in these experiments and the samples were irradiated (~100 W/m^2^ with 10 cm distance) with a halogen lamp (250 W) using a 4 cm water filter to prevent warming of the samples (suspension temperature 22 °C). The compounds studied were dissolved in DMSO due to their limited water solubility. The applied DMSO concentration (up to 4%) did not affect the photochemical activity in the spinach chloroplasts. The inhibitory efficiency of the compounds studied was expressed by IC_50_ values, *i.e.*, by the molar concentration of the compounds that caused a 50% decrease in the oxygen evolution rate relative to the untreated control. The comparable IC_50_ value for a selective herbicide 3-(3,4-dichlorophenyl)-1,1-dimethylurea, DCMU (Diurone^®^) was about 1.9 μmol/L. The results are summarized in Table 3.

### 3.6. *In Vitro* Antifungal Susceptibility Testing

The broth microdilution test [53,54,55] was used for the assessment of the *in vitro* antifungal activity of the synthesized compounds against *Candida albicans* ATCC 44859 (CA), *Candida tropicalis* 156 (CT), *Candida krusei* ATCC 6258 (CK), *Candida glabrata* 20/I (CG), *Trichosporon beigelii* 1188 (TB), *Aspergillus fumigatus* 231 (AF), *Absidia corymbifera* 272 (AC), and *Trichophyton mentagrophytes* 445 (TM). Fluconazole (FLU) was used as the standard since it is a clinically used antimycotic drug. The procedure was performed using a twofold dilution of the compounds in RPMI 1640 (Sevapharma a.s., Prague, Czech Republic) buffered to pH 7.0 with 0.165 mol of 3-morpholino-propane-1-sulfonic acid (MOPS, Sigma, Prague, Czech Republic). The final concentrations of the compounds ranged from 500 to 0.975 μmol/L. Drug-free controls were included. The determination of minimum inhibitory concentration (MIC) was performed according to the Clinical and Laboratory Standards Institute (CLSI, formerly NCCLS) reference protocol M27-A2 for yeasts (IC_80_ value) and M38-A for moulds (IC_50_ value). IC_80_ and IC_50_ were defined as an 80% resp. 50% or greater reduction in growth in comparison with the control. The values of MICs were determined after 24 and 48 h of static incubation at 35 °C. The final MICs for *T. mentagrophytes*, were determined after 72 and 120 h of incubation. The results are summarized in Table 3.

### 3.7. Cell Culture and *in Vitro* Antiproliferative Activity

The human colon cancer cell line HCT116 with the wild type p53 gene (p53^+/+^) were obtained from Maria Sklodowska-Curie Memorial Cancer Center in Gliwice, Poland. Briefly, the cells were seeded in 96-well plates (3 × 10^3^ cells/well) in cellular proliferation assays 24 h before the addition of the chelators. The assay was performed following a 72 h incubation with varying concentrations of the agents. Each compound was tested in triplicate in a single experiment with each experiment being repeated 3 times. After 72 h incubation with the compounds being investigated, 20 µL of MTS solution was added to each well (100 µL) and incubated for 1 h at 37 °C. The optical densities of the samples were analyzed at 490 nm. Results were expressed as a percentage of the control. The inhibitory concentration (IC_50_) was defined as the compound concentration necessary to reduce the absorbance to 50% of the untreated control. The results are summarized in Table 3. 

## 4. Conclusions

A series of fifteen substituted benzylidenethiosemicarbazones and twelve substituted quinolinylvinylthiosemicarbazones were designed, prepared and characterized. The synthesis of these compounds was carried out in microwave conditions, which improves the yield and purity of products isolated. The prepared compounds were tested for their ability to inhibit photosynthetic electron transport (PET) in spinach chloroplasts (*Spinacia oleracea* L.), for their antifungal activity and for their anticancer activity against human colon cancer. All of the compounds were primarily designed as potential thiosemicarbazone-based iron chelators. All of the compounds discussed showed moderate PET inhibition as well as an antifungal effect. (*E*)-2-(quinolin-2-ylvinyl)*-N*,*N*-dimethylhydrazine-carbothioamide (**4b**) expressed the strongest activity among all of the compounds tested; it was comparable to or more active than fluconazole against *Aspergillus fumigatus*, *Absidia corymbifera*, *Candida tropicalis* and *Candida krusei*. Compounds **4b** and **4e** expressed micromolar antiproliferative activity better than standard doxorubicin. According to the results, the quinoline nucleus and the *N*,*N*-dimethylhydrazinecarbothioamide chain can be considered as basic structural fragments for high antiproliferative and antifungal activity. Iron chelation was assumed in UV-VIS spectra analysis. According to the results we can concluded that iron chelating ability of the compounds is one of factors necessary for the activity.

## Supplementary Materials

Supplementary materials can be accessed at: http://www.mdpi.com/1420-3049/17/11/13483/s1.
