# Supplementary material for: Investigation of the Biological Properties of (Hetero)Aromatic Thiosemicarbazones
Source: Molecules. 2012 Nov 14;17(11):13483–502. doi: 10.3390/molecules171113483 (PMC6268061; doi:10.3390/molecules171113483)

Supplementary Materials

Isosbestic point determination for the system iron chelator (Fe^{3+}). Few active and inactive compounds were selected for this comparison. Compounds were dissolved in DMSO and mixed with different amounts of iron chloride to obtain different saturation of ligands with the metal particles. Absorption spectra were collected with microplate reader Synergy (Biotek). To 100 µL of compound in DMSO (1×10^{-3} M) was added $FeCl_3$ to obtain the concentration ratios as shown on figures. Spectra were collected and plotted overlaid to observe isosbestic points. If compound is potent chelator then changes in absorbance allow to noted isosbestic points as in case of **4j** (Figure 1) and **4b** (Figure 2). On the other hand compounds with lower affinity to metal cations give spectra with less visible points (**4i**) (Figure 3). Compounds that should be regarded as inactive (**3a**, **3m**) (Figures 4 and 5) does not interact with metal cations.

Figure 1. Isosbestic analysis of complexes of **4j** with iron 330 nm, 385nm.

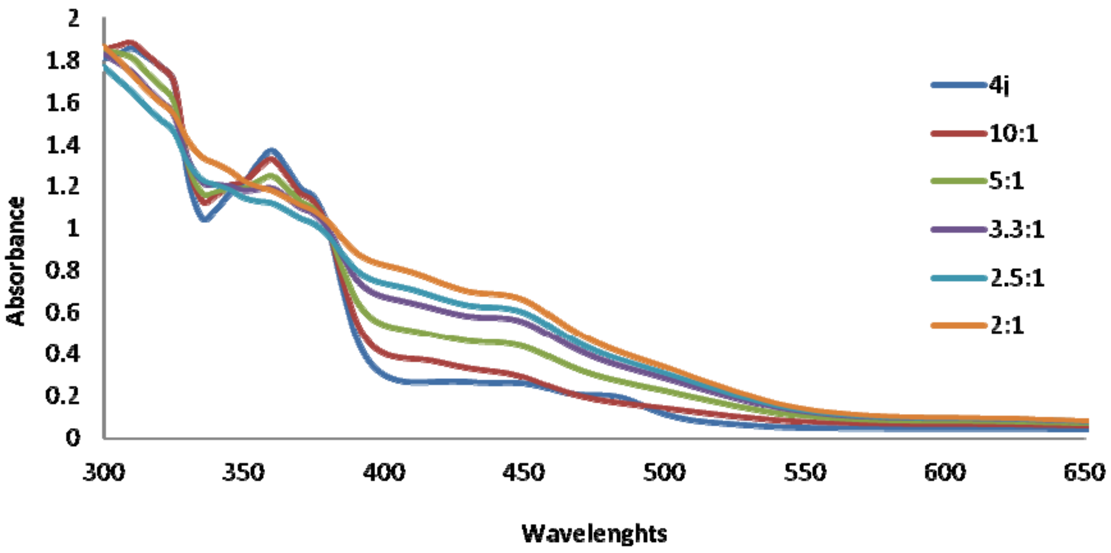

Figure 2. Isosbestic analysis of complexes of **4b** with iron: 375nm.

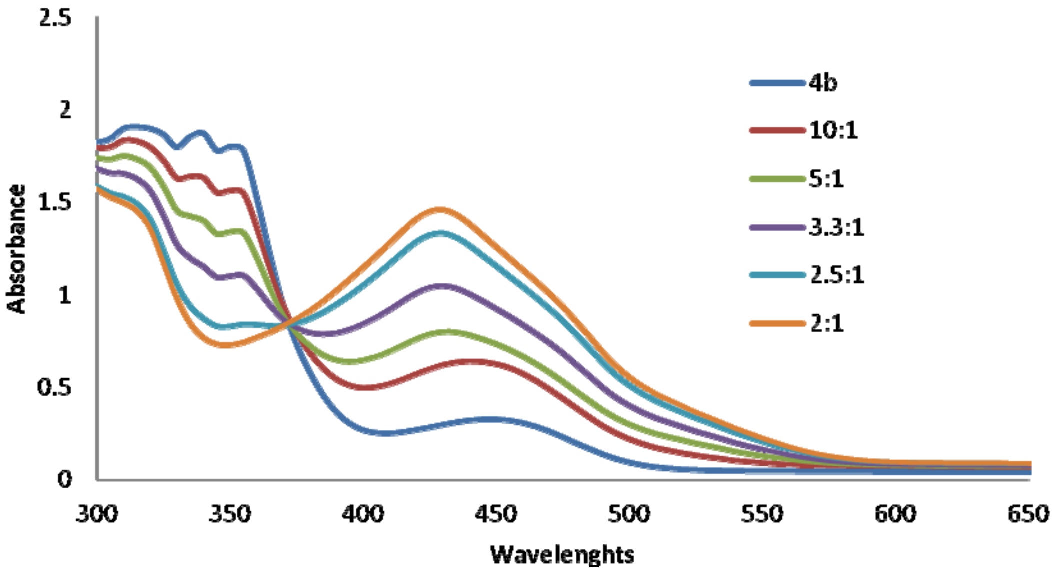

Figure 3. Isosbestic analysis of complexes of **4i** with iron.

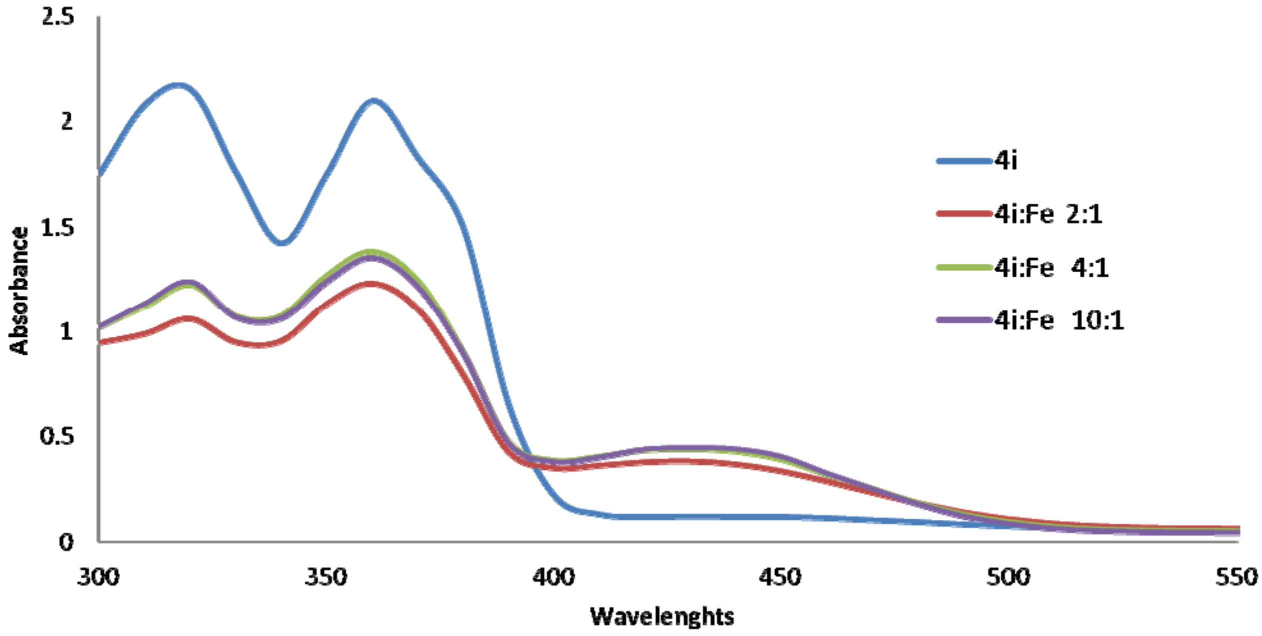

Figure 4. Isosbestic analysis of complexes of **3a** with iron.

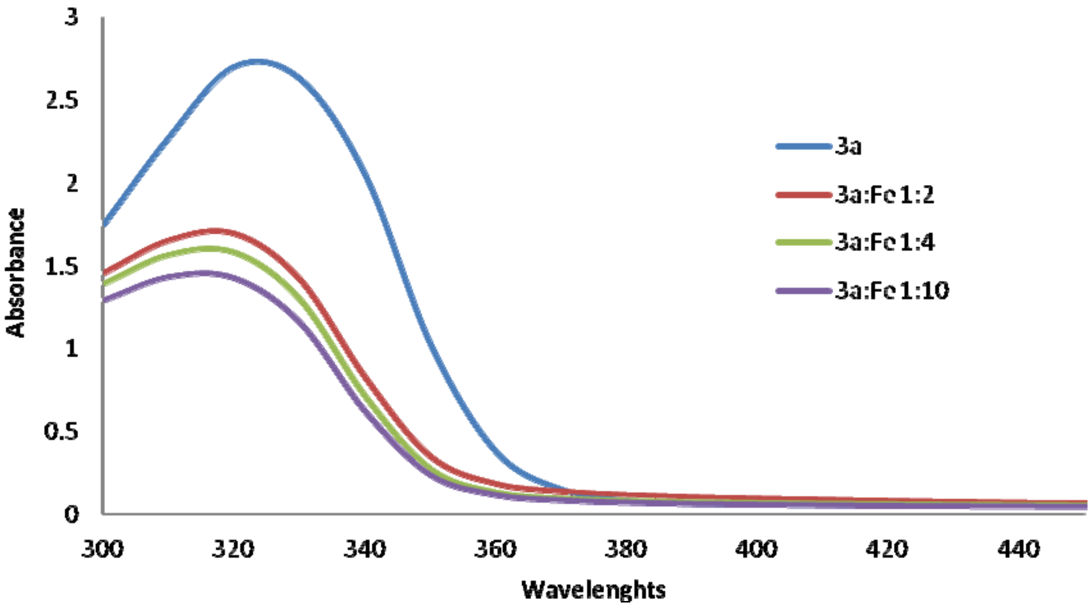

Figure 5. Isosbestic analysis of complexes of **3m** with iron.

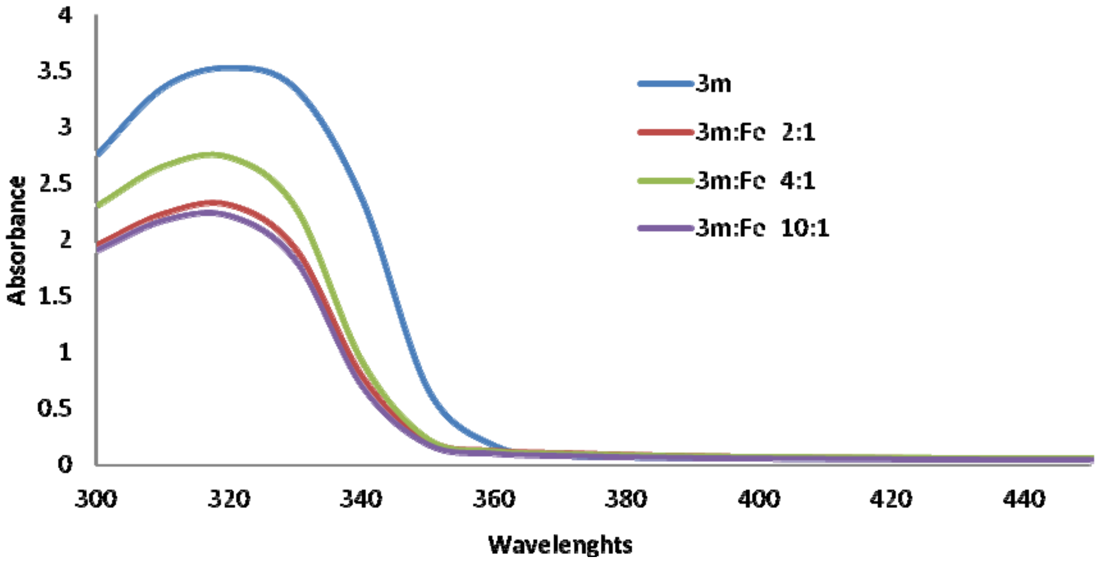

As the aromatic thiosemicarbazones can be considered as Schiff bases they couldexist as *E*- and *Z*-isomeric forms or as a mixture of both isomers. In some cases the presence of selected isomer, mainly *Z*-isomer, could be promoted by intramolecular hydrogen bonding. In other cases the steric hindrance is a main factor in equilibrium between isomers. The most important signal in the process of describing the correct stereoisomer is that connected with NH group. Compounds possessing the *Z*-isomer generally exhibit the NH signal in the 14–15 ppm range, whereas those having an *E*-isomer exhibit the signal in the 9–12 ppm range. To confirm *E*-stereoisomer we carried out NOESY experiment which shows **CH** and **NH** correlation by space.

NMR Experimental: ^{1}H-NMR, COSY and two dimensional ^{1}H-^{1}H NOESY spectra were recorded on a BRUKER AVANCE 500 MHz spectrometer (Figures 6–9). NOESY spectra for compound **3i** was recoded using mixing time value (τ_m) 300ms.

Figure 6. ^{1}H-NMR spectra of **3i**.

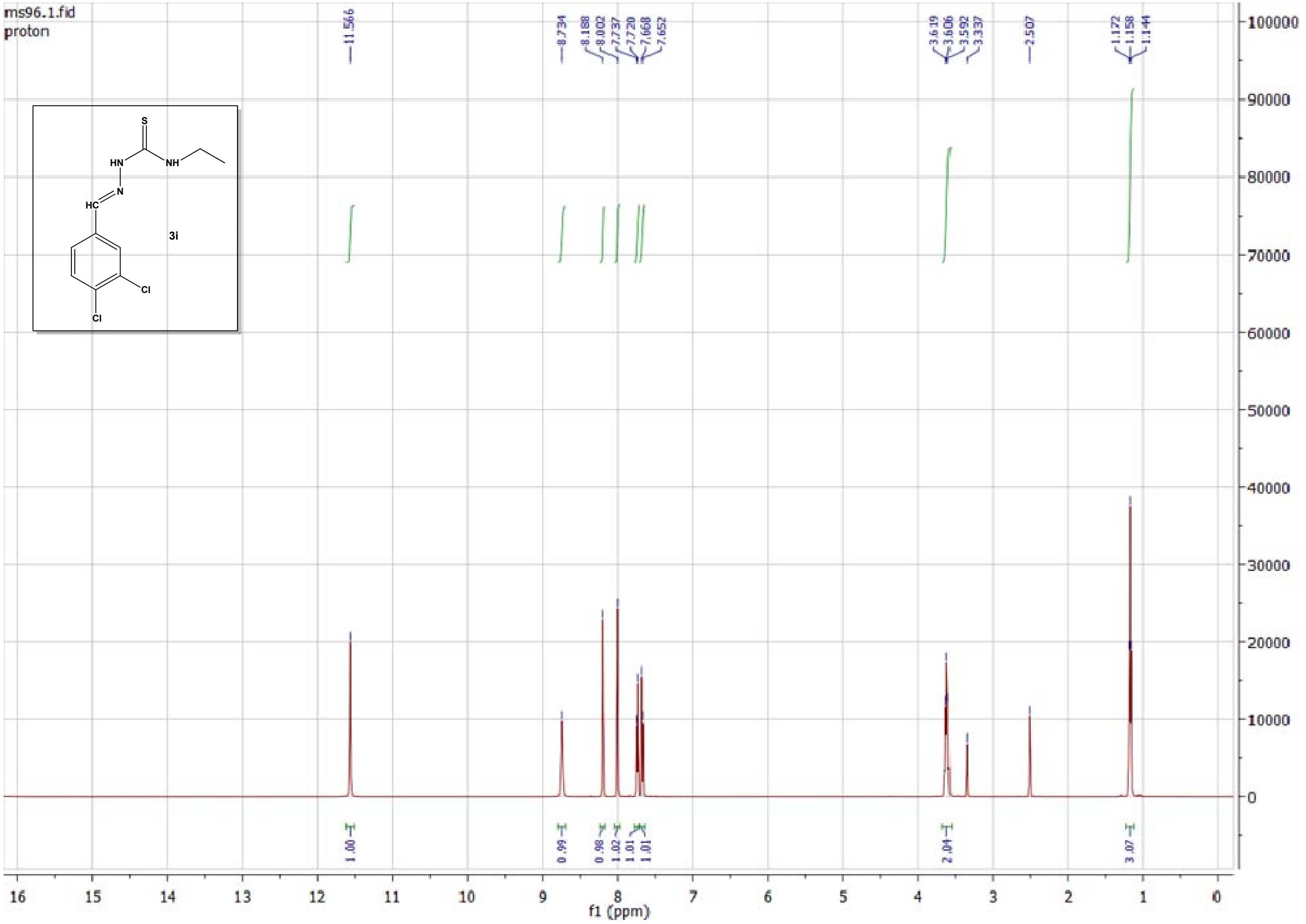

Figure 7. COSY experiment for **3i**.

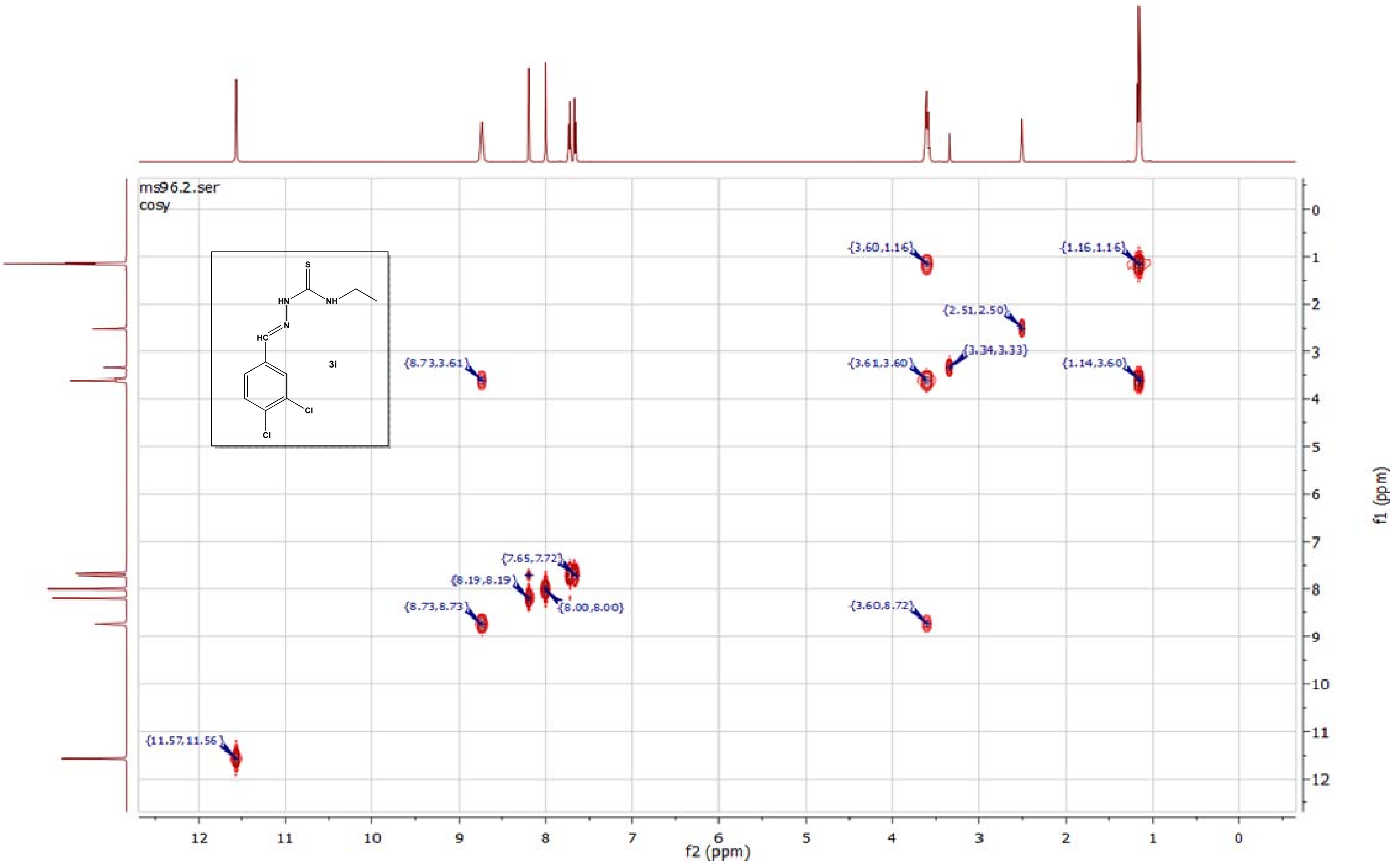

Figure 8. NOESY experiment for **3i**.

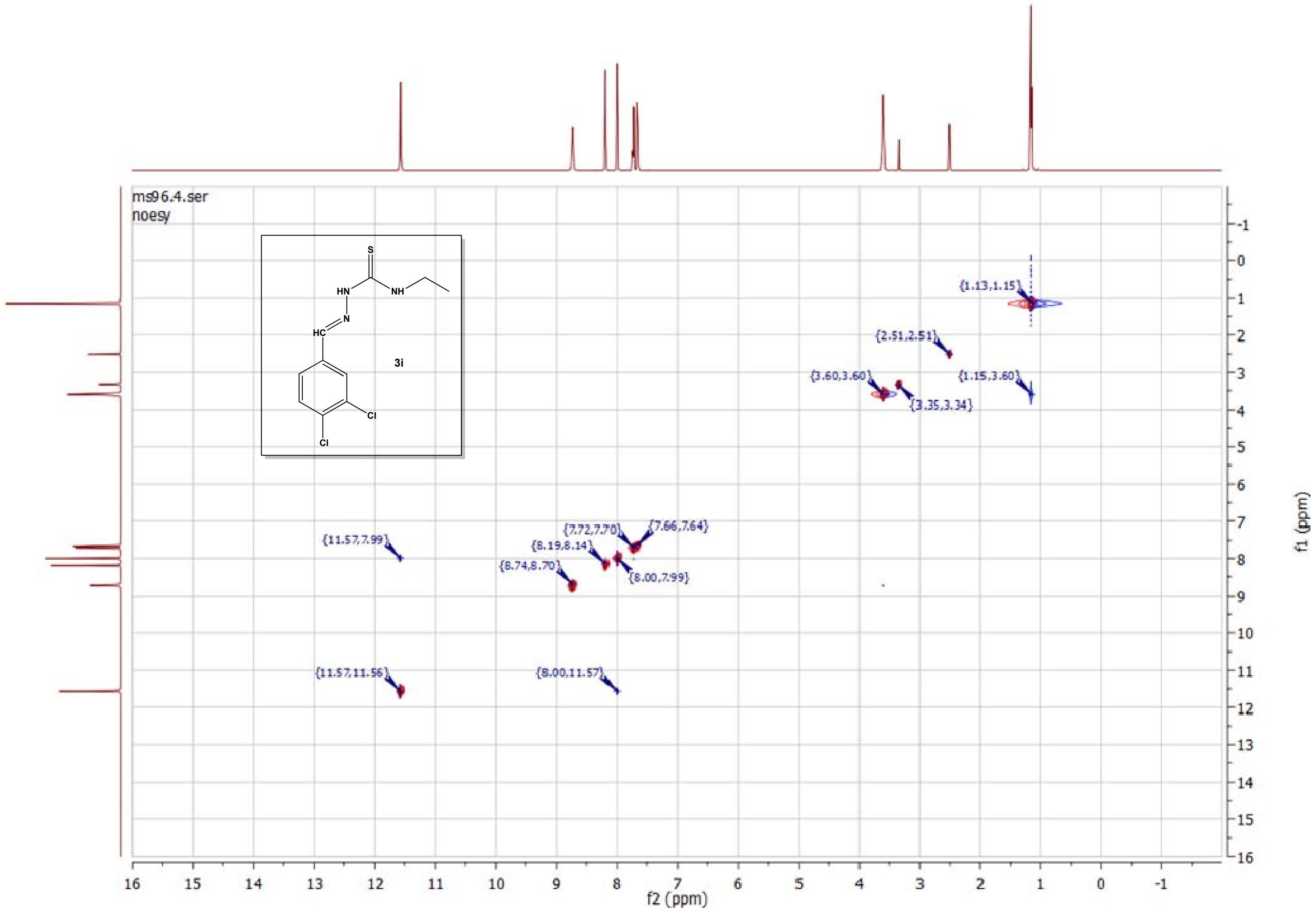

Figure 9. NOESY cropped for the CH-NH correlation in **3i**.

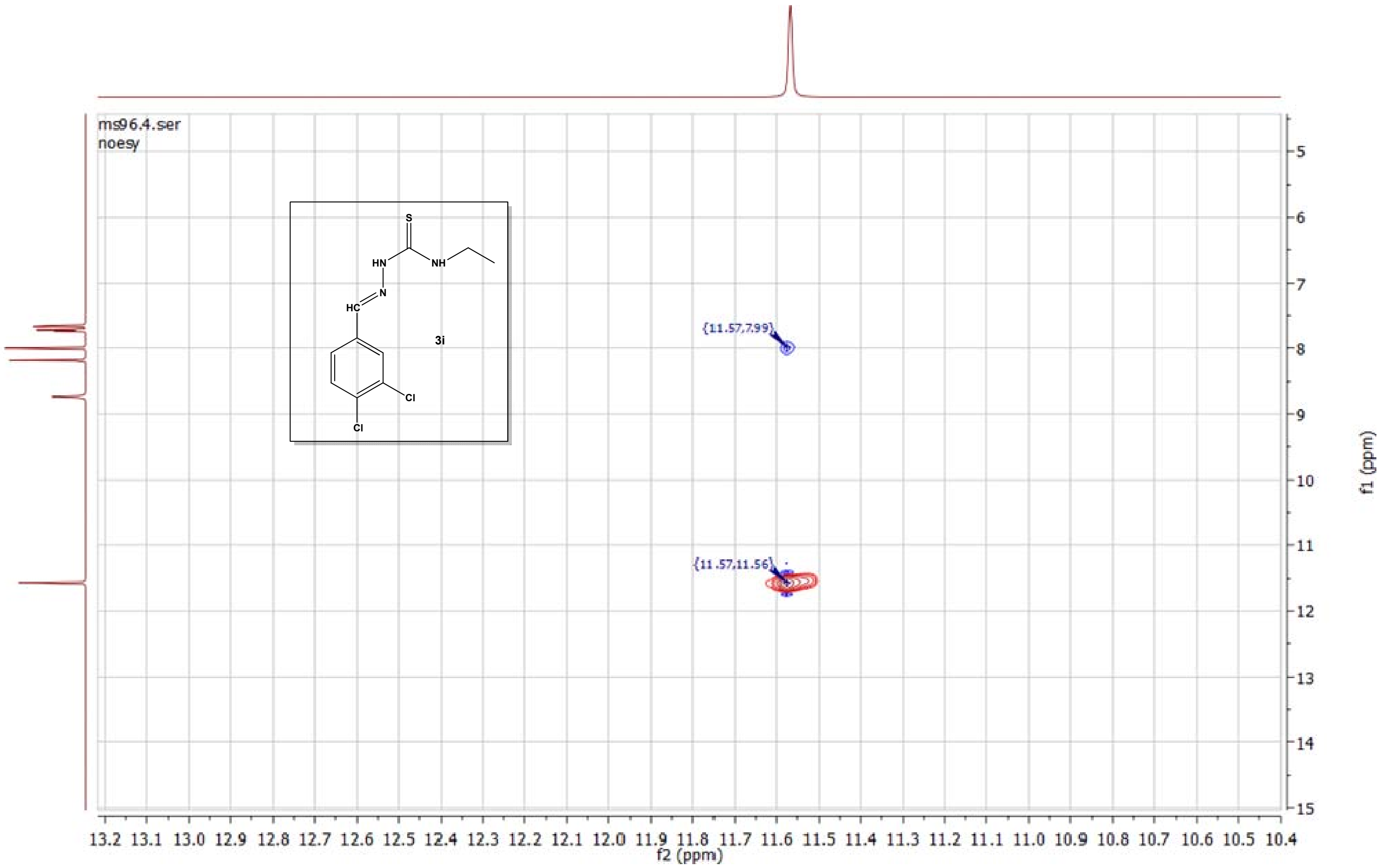

Supplement: Supplementary file 1 [file molecules-17-13483-s001.pdf]
